# Early Doppler-echocardiography evaluation of Carpentier-Edwards Standard and Carpentier-Edwards Magna aortic prosthetic valve: comparison of hemodynamic performance

**DOI:** 10.1186/1476-7120-9-37

**Published:** 2011-11-24

**Authors:** Giovanni Minardi, Giovanni Pulignano, Donatella Del Sindaco, Martina Sordi, Herribert Pavaci, Amedeo Pergolini, Giordano Zampi, Francesca Moschella Orsini, Carlo Gaudio, Francesco Musumeci

**Affiliations:** 1 Department of Cardiovascular Science, "S. Camillo-Forlanini" Hospital, Rome, Italy; 2 Department "Heart and Great Vessels Attilio Reale", Sapienza University of Rome, Rome, Italy

**Keywords:** Carpentier-Edwards, Doppler-echocardiography, prosthetic aortic valve

## Abstract

**Objectives:**

This study was designed to describe Doppler-echocardiography values of Carpentier-Edwards Perimount Standard (CEPS) and Carpentier-Edwards Perimount Magna (CEPM) aortic prosthetic valves, evaluated by a single, experienced echo-laboratory, early in the postoperative phase.

**Methods:**

Three-hundred-seventy-seven consecutive patients, who had had a CEPS or a CEPM implanted in our Hospital due to aortic stenosis and/or insufficiency, underwent baseline Doppler echocardiography evaluation within 7 days after surgery. Hemodynamic performances of CEPS and CEPM were accurately described, evaluating flow-dependent (transprosthetic velocities and gradients) and flow-independent (effective orifice area, indexed effective orifice area and Doppler velocity index) Doppler-echocardiography parameters.

**Results:**

Out of the 377 patients 48.8% were men (n = 184), mean age was 74.63 ± 6.77 years, mean BSA was 1.78 ± 0.18 m2, mean ejection fraction was 57.78 ± 8%. Two-hundred and sixty two CEPS and 115 CEPM were implanted. Comparing size-by-size CEPS with CEPM, both prostheses showed a good hemodynamic profile, with fairly similar values of pressure gradients (PGmax and mean, in mmHg, = 37,18 ± 11.57 and 20.81 ± 7.44 in CEPS n°19 compared to 32,47 ± 7,76 and 17,67 ± 4.63 in CEPM n°19 and progressively lower in higher sized prostheses, having PGmax and mean 15 ± 3,16 and 9.15 ± 1,29 in CEPS n°29 compared to 15,67 ± 1,53 and 9 ± 1 in CEPM n°29) and EOAi (being 0,65 ± 0,33 cm²/m² in CEPS n°19 compared to 0,77 ± 0,29 cm²/m² in CEPM n°19 and progressively higher in higher sized prostheses, being 1,28 ± 0,59 cm²/m² in CEPS n°29 compared to 1,07 ± 0,18 cm²/m² in CEPM n°29), the latter resulting, however, basically less flow obstructive.

**Conclusions:**

Our data confirm the good hemodynamic performance of both aortic bioprostheses and the more favourable hemodynamic profile of CEPM compared to CEPS, pointing out the need to perform routinely an accurate baseline Doppler-echocardiography evaluation early after surgery to allow an adequate interpretation of data at follow-up.

## Introduction

Doppler-echocardiography is widely used to study the hemodynamic performance of prosthetic aortic valves. As for native valves, several Doppler echocardiography parameters (i.e. pressure gradients, effective orifice area, Doppler velocity index) can be estimated for prosthetic valves, but the interpretation of the data is much more difficult. Thus, the assessment of normal and abnormal function of heart valve prostheses remains challenging [1-6].

One of the main problems is that prosthetic valves are, to some degree, obstructive to blood flow. This makes it difficult to decide whether a calculated Doppler-echocardiography measure represents the performance of a normal functioning valve or whether it indicates prosthetic valve dysfunction [4-10]. The valve type and size play an important role in determining hemodynamic features and therefore an adequate interpretation of Doppler-echocardiography data requires the knowledge of the exact type and size of the implanted valve. Certainly, the development of tables based on solid data summarizing normal value range would be useful to evaluate the Doppler-echocardiography measurements in each patient [4-6].

The hemodynamic performance of aortic prostheses is attracting new interest due to the influence of patient-prosthesis-mismatch (PPM) on left ventricular mass regression and on clinical outcome after valve replacement (AVR) [11,12].

Several studies have tried to give an overview of available data, but they have been limited by insufficient patient numbers, different timings of the Doppler-echocardiography evaluation, the large number of valve types available on the market, and multicenter echocardiographic assessment [4,5].

The aim of our study was to define, early in the postoperative phase, the Doppler-echocardiography hemodynamic performance of the bioprosthetic Carpentier-Edwards Perimount standard (CEPS) aortic valves compared to Carpentier-Edwards Perimount Magna (CEPM). The study was performed in a single, experienced echo-laboratory. The incidence of PPM, defined as an effective orifice area indexed (EOAi) <0,85 cm²/m² [11], was also evaluated. A clinical and Echo follow-up has been programmed in all patients with the aim to collect further data useful for the interpretation and clinical implications of echocardiography results.

## Methods

Between January 2007 and October 2010, 377 consecutive patients affected by aortic stenosis (AS) and/or aortic insufficiency (AI), who had had a CEPS (n = 262) or a CEPM (n = 115) implanted in our Hospital were recluted for this study. This study was approved by our local ethics committee and informed consent was obtained from all patients.

Indications for aortic valve replacement were: hemodynamically severe AS, severe AI or moderate AS associated with coronary artery disease requiring surgical revascularization. Patients undergoing an isolated AVR or those requiring AVR associated with aorto-coronary bypass grafting (CABG) were included in the study. Patients with concomitant mitral valve replacement were excluded from the study.

All patients underwent complete preoperative Doppler-echocardiography evaluation and a control Doppler-echocardiography examination within 7 days after surgery, following American Society of Echocardiography guidelines [13]. The Doppler-echocardiography examinations were performed with a iE33 (Philips, Eindhoven, The Netherlands) in the same echo-laboratory by four senior echocardiographers. The investigator was blinded to the prosthetic valve type and size.

Great attention was paid to the assessing of left ventricle outflow tract (LVOT), the most observer-dependent variable in EOA assessment [14]. The LVOT diameter was measured and used in single patient for calculating EOA and EOAi before and after surgery.

All patients were investigated with the use of color-Doppler, as well as PW and CW Doppler.. The velocity profiles were traced along the outer border of the spectral display. The velocity time integral (VTI) of PW Doppler recordings (sample size 5 mm) from the LVOT (VTI LVOT) and the VTI of CW Doppler recordings of the highest transprosthetic velocity (VTI Ao) were evaluated and Doppler velocity index (DVI = VTI LVOT/VTI Ao) was calculated.

The maximum flow velocity (Vmax), maximum (PGmax) and mean (PGmean) transprosthetic gradients, EOA, indexed EOA (EOAi), and PPM were calculated. The mean of three Doppler measurements in patients with sinus rhythm and the mean of 5 measurements in patients with atrial fibrillation were calculated. The presence of physiological intraprosthetic regurgitation and/or paraprosthetic leak was investigated.

Left ventricle ejection fraction (EF) was estimated through Simpson's method.

A clinical and baseline Echo follow-up has been programmed in all patients; stress echocardiography was scheduled in patients with persistent features of PPM, to evaluate the functional hemodynamic performance of prosthetic aortic valve.

### Valve design and surgical procedure

The CEPS aortic valve bioprosthesis (Edwards Lifesciences, Irvine, California, USA) is a low-profile trileaflet valve composed of bovine pericardium preserved in a buffered glutaraldehyde solution and mounted on a flexible frame. CEPS available sizes for the aortic position range from 19 mm to 29 mm; all of these sizes were used in this study,

The CEPM aortic xenograft is a modification of the CEPS valve. Firstly, the width of the sewing ring has been significantly reduced so that the external diameter is 2 mm smaller than the corresponding CEPS valve. Secondly, the sewing cuff has been displaced upstream, so both sewing cuffs and leaflets remain in a complete supra-annular position, thus achieving a maximal clearance of the aortic valve orifice. Thirdly, the sewing cuff is more flexible and scalloped, thereby facilitating the valve seating and decreasing risk of dehiscence [15].

All patients underwent aortic valve replacement through conventional midline sternotomy, during total normothermic cardiopulmonary bypass. Myocardial protection was achieved by intermittent anterograde warm blood cardioplegia. The aorta was opened, the native aortic valve was excised and complete removal of calcium from the annulus was performed. Prosthesis size was selected according to the size of the aortic annulus, which was incasured using specific manufacturer's sizers. Double armed 2-0 Ethibond sutures were passed through aortic annulus and valve ring. Once all the sutures were placed, the prosthesis was slid down to the supraannular position and the sutures were tied. The aortotomy was closed using 3-0 polypropylene suture stitches with an over-and-over technique.

### Statistical analysis

The continuous variables were expressed as mean values ± standard deviations (SD) and compared using a t-test and the Mann-Whitney U-test as indicated. Categorical variables are presented as frequencies and percentages and compared by Pearson's χ2 test with continuity correction or 2-sided Fisher exact test as appropriate. Statistical significance was defined as p-value < 0,05. SPSS application software version 11.5 (SPSS Inc. Chicago, Ill) was used to perform the statistical analysis.

## Results

### Study population

Three hundred seventy seven patients with AS and/or AI were studied. Out of these patients 48,80% were men (n = 184). Mean age was 74,63 ± 6,77 years. Mean BSA was 1,78 ± 0,18 m2. Mean EF was 57,78 ± 8,00% in whole study population.

CEPS was implanted in 262 patients (69,49%) and CEPM in 115 patients (30,51%), respectively. The indication for aortic valve replacement was severe AS in 322 patients (85.41%). Forty-eight patients (14,9%) with AS underwent CABG and valve replacement, 29 of whom had a CEPS implanted. There were no statistically significant differences in the preoperative clinical characteristics between CEPS and CEPM populations (Table 1).

**Table 1:** Population characteristics

	CEPS (n = 262)	CEPM (n = 115)	*p value*
Mean Age (years)	74,71 ± 7,16	74,46 ± 5,95	0,89

Gender (Male)	126	58	0,236

Mean BSA (m²)	1,78 ± 0,18	1,79 ± 0,18	0,718

Mean EF (%)	57,66 ± 9,03	58,05 ± 8,94	0,355

LVEF < = 40%	9	1	0,177

Hypertension	123	58	0,217

Diabetes	23	12	0,476

Dislipidemia	45	21	0,588

Smoke	34	15	0,799

Atrial Fibrillation	23	12	0,476

Familiarity	24	10	0,964

Obesity	47	20	0,877

**Therapy**			

β-blockers	93	34	0,48

Calcium antagonists	63	27	0,824

ACE inhibitors	110	51	0,333

Nitrates	5	1	0,5

Diuretics	147	62	0,775

Statins	25	9	0,725

Others	140	59	0,821

**Aortic valve lesion**			

Stenosis	89,70%	75,70%	

Insufficency	3,80%	8,70%	

Mixed	6,10%	6,08%	

AVR and CABG	0,40%	9,52%	

### Carpentier-Edwards Perimount standard (CEPS)

Out of 262 CEPS, 126 were implanted in men (48,09%). Mean age of patients was 74,71 ± 7,16 years, mean EF was 57,66 ± 9,03%, mean BSA was 1.78 ± 0.18 m2. Hemodynamic parameters for each size of CEPS population are described in Table 2. The presence of physiological intra-prosthetic regurgitation was found in 10% patients; no patient showed signs of para-prosthetic leak.

**Table 2:** EarlyDoppler-Echocardiography evaluation of CEPS

Size	n	BSA (m²)	LVOT (mm)	PG max (mmHg)	PG mean (mmHg)	DVI	EOA (cm²)	EOAi (cm²/m²)
**19**	101	1,63 ± 0,4	18,42 ± 2,15	37,18 ± 11,57	20,81 ± 7,44	0,42 ± 0,10	1,15 ± 0,50	0,65 ± 0,33

**21**	76	1,76 ± 0,19	18,54 ± 3,75	31,00 ± 8,90	16,85 ± 5,31	0,48 ± 0,15	1,37 ± 0,41	0,79 ± 0,23

**23**	58	1,84 ± 0,16	20,91 ± 3,71	27,24 ± 12,07	14,05 ± 6,04	0,45 ± 0,09	1,60 ± 0,41	0,89 ± 0,27

**25**	19	1,89 ± 0,15	21,45 ± 2,04	24,50 ± 7,67	12,87 ± 4,09	0,43 ± 0,12	1,52 ± 0,39	0,81 ± 0,23

**27**	4	1,91 ± 0,19	25,50 ± 1,29	21,50 ± 6,76	10,67 ± 6,30	0,40 ± 0,08	1,98 ± 0,28	1,1 ± 0,26

**29**	4	2,10 ± 0,23	27,50 ± 1,29	15,00 ± 3,16	9,15 ± 1,29	0,44 ± 0,05	2,55 ± 1,26	1,28 ± 0,59

### Carpentier-Edwards Perimount Magna (CEPM)

Out of 115 CEPM, 58 were implanted in men (50,43%). Mean age of patients was 74,46 ± 5,95 years, mean EF was 58,05 ± 8,94%, mean BSA was 1.79 ± 0.18 m2. Hemodynamic parameters for each size of CEPM population are described in Table 3. The presence of physiological intra-prosthetic regurgitation was found in 9% patients; no patient showed signs of para-prosthetic leak.

**Table 3:** Early Doppler-Echocardiography evaluation of CEPM

Size	n	BSA (m²)	LVOT (mm)	PG max (mmHg)	PG mean (mmHg)	DVI	EOA (cm²)	EOAi (cm²/m²)
**19**	30	1,59 ± 0,15	18,07 ± 1,86	32,47 ± 7,76	17,67 ± 4,63	0,46 ± 0,14	1,22 ± 0,49	0,77 ± 0,29

**21**	43	1,77 ± 0,16	19,41 ± 2,24	28,93 ± 9,16	15,79 ± 5,01	0,60 ± 0,46	1,42 ± 0,59	0,80 ± 0,32

**23**	24	1,90 ± 0,18	20,49 ± 2,61	23,90 ± 7,06	13,04 ± 4,40	0,48 ± 0,12	1,64 ± 0,42	0,79 ± 0,36

**25**	13	1,89 ± 0,13	22,17 ± 2,71	26,36 ± 6,90	14,18 ± 3,40	0,49 ± 0,14	2,11 ± 0,58	1,12 ± 0,36

**27**	2	1,93 ± 0,04	22,50 ± 3,53	14,00 ± 1,41	5 ± 4,24	0,71	2,45 ± 0,21	1,27 ± 0,14

**29**	3	1,86 ± 0,12	26,33 ± 2,89	15,67 ± 1,53	9 ± 1	0,37 ± 0,01	1,99 ± 0,36	1,07 ± 0,18

### Comparison between CEPS and CEPM

The hemodynamic data of CEPS and CEPM patients, excluding those patients with PPM, showed similar values of GP max and mean, EOA and EOAi, overlapping substantially with Literature data (Additional files 1 &2). The size-by-size hemodynamic parameters of the two groups are shown in Table 4.

**Table 4:** Comparison between CEPS and CEPM

		CEPS n° 91	CEPM n°66	P value
**19**		19	11	

	**PG max (mmHg)**	37,18 ± 11,57	32,47 ± 7,76	0,12

	**DVI**	0,42 ± 0,10	0,46 ± 0,14	0,11

	**EOAi (cm²/m²)**	0,65 ± 0,33	0,77 ± 0,29	0,12

**21**		27	24	

	**PG max (mmHg)**	31,00 ± 8,90	28,93 ± 9,16	0,34

	**DVI**	0,48 ± 0,15	0,60 ± 0,46	0,038

	**EOAi (cm²/m²)**	0,79 ± 0,23	0,80 ± 0,32	0,7

**23**		26	15	

	**PG max (mmHg)**	27,24 ± 12,07	23,90 ± 7,06	0,22

	**DVI**	0,45 ± 0,09	0,48 ± 0,12	0,05

	**EOAi (cm²/m²)**	0,89 ± 0,27	0,79 ± 0,36	0,43

**25**		11	11	

	**PG max (mmHg)**	24,50 ± 7,67	26,36 ± 6,90	0,85

	**DVI**	0,43 ± 0,12	0,49 ± 0,14	0,16

	**EOAi (cm²/m²)**	0,81 ± 0,23	1,12 ± 0,36	0,027

**27**		4	2	

	**PG max (mmHg)**	21,50 ± 6,76	14 ± 1,41	0,22

	**DVI**	0,40 ± 0,08	0,71	0,84

	**EOAi (cm²/m²)**	1,1 ± 0,26	1,27 ± 0,14	0,47

**29**		4	3	

	**PG max (mmHg)**	15,00 ± 3,16	15,67 ± 1,53	0,75

	**DVI**	0,44 ± 0,05	0,37 ± 0,01	0,18

	**EOAi (cm²/m²)**	1,28 ± 0,59	1,07 ± 0,18	0,59

### Incidence of PPM

In CEPS group the cumulative incidence of PPM was 65,26% and resulted higher than in the CEPM group (42,60%), significantly (p value <0,0001). The incidence of PPM was inversely correlated to prosthesis size in both groups (Table 5). However, in CEPM we noticed a trend towards lower gradients and higher area (Table 6 and 7).

**Table 5:** Prothesis Mismatch

Size	CEPS	CEPM	P Value
**19**	82 (81,18%)	19 (63,33%)	0,0001

**21**	49 (64,47%)	19 (44,18%)	0,0001

**23**	32 (55,17%)	9 (37,5%)	0,0001

**25**	8 (42,10%)	2 (15,38%)	0,0001

**27**	0	0	

**29**	0	0	

**Cumulative**	171 (65,26%)	49 (42,60%)	

**Table 6:** Comparison of Hemodynamics Performance between CEPS and CEPM with and without PPM

		CEPS (N°262)		P value	CEPM (N°115)		P value
**19**		**PPM (82)**	**No PPM (19)**		**PPM (19)**	**No PPM (11)**	

	**PG max (mmHg)**	36,97 ± 12,29	38,05 ± 7,99	0,72	34,65 ± 7,45	29,36 ± 7,31	0,077

	**PG mean (mmHg)**	20,65 ± 7,86	21,47 ± 5,36	0,67	18,69 ± 4,54	14,60 ± 2,84	0,018

	**DVI**	0,39 ± 0,08	0,53 ± 0,12	0,0001	0,49 ± 0,13	0,59 ± 0,08	0,017

	**EOAi (cm²/m²)**	0,58 ± 0,12	1,13 ± 0,44	0,0001	0,71 ± 0,39	1,03 ± 0,40	0,005

**21**		**PPM (49)**	**No PPM (27)**		**PPM (19)**	**No PPM (24)**	

	**PG max (mmHg)**	31,79 ± 8,72	29,56 ± 9,20	0,30	31,15 ± 12,78	28,42 ± 10,95	0,46

	**PG mean (mmHg)**	17,43 ± 5,09	15,81 ± 5,64	0,22	17,36 ± 6,24	15,95 ± 5,52	0,43

	**DVI**	0,42 ± 0,09	0,59 ± 0,18	0,0001	0,59 ± 0,47	0,60 ± 0,26	0,92

	**EOAi (cm²/m²)**	0,65 ± 0,11	1,04 ± 0,19	0,0001	0,65 ± 0,18	1,03 ± 0,28	#####

**23**		**PPM (32)**	**No PPM (26)**		**PPM (9)**	**No PPM (15)**	

	**PG max (mmHg)**	27,86 ± 12,86	26,46 ± 11,22	0,66	22,11 ± 7,04	24,28 ± 7,61	0,49

	**PG mean (mmHg)**	14,97 ± 6,99	12,92 ± 4,50	0,20	12, 00 ± 4,80	13,36 ± 4,53	0,50

	**DVI**	0,40 ± 0,07	0,51 ± 0,9	0,0001	0,63 ± 0,45	0,52 ± 0,12	0,39

	**EOAi (cm²/m²)**	0,71 ± 0,11	1,16 ± 0,20	0,0001	0,85 ± 0,43	1,03 ± 0,21	0,20

**25**		**PPM (8)**	**No PPM (11)**		**PPM (2)**	**No PPM (11)**	

	**PG max (mmHg)**	28,25 ± 10,82	23,90 ± 6,52	0,30	36,00 ± 9,89	20, 55 ± 8,38	0,23

	**PG mean (mmHg)**	14,63 ± 5,55	12,80 ± 4,10	0,43	18,00 ± 4,24	10,90 ± 4,31	0,064

	**DVI**	0,32 ± 0,15	0,49 ± 0,06	0,005	0,44 ± 0,11	0,57 ± 0,13	0,26

	**EOAi (cm²/m²)**	0,63 ± 0,13	0,85 ± 0,41	0,17	0,68	1,29 ± 0,40	0,22

**Table 7:** Comparison of Hemodynamics Performance between CEPS and CEPM with and without PPM

		CEPS	CEPM	P value	CEPS	CEPM	P value
**19**		**PPM (82)**	**PPM (19)**		**No PPM (19)**	**No PPM (11)**	

	**PG max (mmHg)**	36,97 ± 12,29	34,65 ± 7,45	0,45	38,05 ± 7,99	29,36 ± 7,31	0,006

	**PG mean (mmHg)**	20,65 ± 7,86	18,69 ± 4,54	0,34	21,47 ± 5,36	14,60 ± 2,84	0,0001

	**DVI**	0,39 ± 0,08	0,49 ± 0,13	0,007	0,53 ± 0,12	0,59 ± 0,08	0,17

	**EOAi (cm²/m²)**	0,58 ± 0,12	0,71 ± 0,39	0,17	1,13 ± 0,44	1,03 ± 0,40	0,55

**21**		**PPM (49)**	**PPM (19)**		**No PPM (27)**	**No PPM (24)**	

	**PG max (mmHg)**	31,79 ± 8,72	31,15 ± 12,78	0,81	29,56 ± 9,20	28,42 ± 10,95	0,69

	**PG mean (mmHg)**	17,43 ± 5,09	17,36 ± 6,24	0,96	15,81 ± 5,64	15,95 ± 5,52	0,93

	**DVI**	0,42 ± 0,09	0,59 ± 0,47	0,017	0,59 ± 0,18	0,60 ± 0,26	0,89

	**EOAi (cm²/m²)**	0,65 ± 0,11	0,65 ± 0,18	0,99	1,04 ± 0,19	1,03 ± 0,28	0,85

**23**		**PPM (32)**	**PPM (9)**		**No PPM (26)**	**No PPM (15)**	

	**PG max (mmHg)**	27,86 ± 12,86	22,11 ± 7,04	0,09	26,46 ± 11,22	24,28 ± 7,61	0,52

	**PG mean (mmHg)**	14,97 ± 6,99	12, 00 ± 4,80	0,24	12,92 ± 4,50	13,36 ± 4,53	0,77

	**DVI**	0,40 ± 0,07	0,63 ± 0,45	0,16	0,51 ± 0,9	0,52 ± 0,12	0,67

	**EOAi (cm²/m²)**	0,71 ± 0,11	0,85 ± 0,43	0,37	1,16 ± 0,20	1,03 ± 0,21	0,095

**25**		**PPM (8)**	**PPM (2)**		**No PPM (11)**	**No PPM (11)**	

	**PG max (mmHg)**	28,25 ± 10,82	36,00 ± 9,89	0,39	23,90 ± 6,52	20, 55 ± 8,38	0,34

	**PG mean (mmHg)**	14,63 ± 5,55	18,00 ± 4,24	0,45	12,80 ± 4,10	10,90 ± 4,31	0,33

	**DVI**	0,32 ± 0,15	0,44 ± 0,11	0,31	0,49 ± 0,06	0,57 ± 0,13	0,15

	**EOAi (cm²/m²)**	0,63 ± 0,13	0,68	0,73	0,85 ± 0,41	1,29 ± 0,40	0,063

## Discussion

The aim of this study was to describe baseline hemodynamic parameters for CEPS and CEPM prosthetic valves early in the postoperative phase. Although it has been shown that an early investigation could differ from a late investigation, it has also been assessed that when prosthetic valve dysfunction is suspected a previous Doppler echocardiography evaluation for comparison can be helpful [6,16]. Thus, an early Doppler-echocardiography assessment of prosthetic valves is of great importance, providing reference hemodynamic parameters useful for an appropriate follow-up. We selected the two biological prosthesis types more frequently used in our Hospital and we systematically evaluated all consecutive patients in which one of them was implanted.

Previous Doppler-echocardiography studies have suggested that velocity measurements are not suitable for detecting less than severe obstruction in prosthetic valves [14,17,18]. Hence, to perform a comprehensive Doppler-echocardiography assessment of the prostheses, we evaluated not only flow-dependent parameters (flow velocities and pressure gradients), but also flow-independent parameters (EOA, EOAI and DVI).

In our study, considering all prostheses, the PG max and mean resulted higher and the EOAi resulted lower than those reported in Literature, in which a lower number of patients were evaluated [15,19,20]. These different results could be explained by the early evaluation of patients, in which a stable hemodynamic condition is not completely recovered, because of anemia etc. Probably, an echocardiographic study, performed at the same time as the reported studies (>15 days), would provide similar results. However, early echocardiographic parameters of only the CEPS and CEPM considered normally functioning and well-matched are similar to those reported in Literature and obtained in the later evaluation during the follow-up. In our study, the DVI, a flow independent parameter, not routinely considered in previous studies, was evaluated, showing all prostheses normally functioning, despite quite abnormal gradients and area values, even in a group with features of PPM.

The consistent number of valves studied and the homogeneity in valve type, in implantation technique, and in Doppler-echocardiography evaluation, considering a flow independent parameter, are the relevant characteristics of this study, giving strength to the hemodynamic reference values described.

Previous evidence, already discussed in Literature, showed a wide range of normality in prosthetic valve hemodynamic parameters [4,5]. Our data confirm this wide range of variability, pointing out the need to perform routinely an accurate evaluation of aortic prosthetic valve hemodynamic profile in the early post-operative phase in each patient. A consistent change of Doppler-echocardiography parameters from the early baseline investigation may be a useful warning sign during follow-up.

To our knowledge no previous studies have compared a large sample of CEPS and CEPM prosthetic valves divided size-by-size, evaluated in the same protocol by a single echo-laboratory, in the early post-operative phase.

Our study did not show any statistically significant differences between in both prostheses PGmax, DVI and EOAi, except for EOAi in 25-size prosthesis and DVI in 21- and 23-size prosthesis. Nevertheless, CEPM had basically lower PG max and mean, and higher EOAi compared to CEPS size-by-size.

Generally, prosthetic valves are inherently stenotic due to the sewing ring as well as the stent itself that may contribute to reducing the existing orifice area and therefore lead to PPM. The influence of PPM on LV mass regression and on clinical outcome after AVR is attracting new interest [11,12]. PPM, as we know, has prognostic implications particularly in the presence of preoperative left ventricular dysfunction, in the lack of left ventricular mass regression and contributes to increased mortality and the number of cardiac events after AVR [21,22]. PPM is a frequent cause of increased trans-prosthetic gradient. It is important to differentiate this condition from acquired prosthetic stenosis which may result from leaflet calcification, pannus overgrowth or thrombus formation. In our study the incidence of PPM was higher than reported in Literature in both prostheses and could reflect the impaired hemodynamics of early post-operative phase [23,24] Several studies have tried to give an overview of available data, but they have been limited by insufficient patient numbers, different timings of the Doppler-echocardiography evaluation, the large number of valve types available on the market, and multicenter echocardiographic assessment [4,5]. Our study was performed in a single, experienced echo-laboratory. The incidence of PPM, defined as EOAi <0,85 cm²/m² was evaluated according Literature criteria [11]. A DVI, derived from the ratio between VTI LVOT and the VTI Ao, was measured as further index of well functioning aortic prosthetic valve, similar to a native valve, assuming as normal value a DVI >0.40. As reported in the methods section, a late clinical and echocardiographic evaluation of all the patients has been programmed with the aim of detecting the true PPM from the transient, apparent, early one.

The following conditions were identified in the early evaluation: patients with EOAi >0.85 cm²/m² and DVI>0.40 (n° 130: CEPS n°85, CEPM n°45), patients with EOAi <0.85 cm²/m² and DVI>0.40 (n° 141: CEPS n°101, CEPM n° 40) and patients with EOAi <0.85 cm²/m² and DVI<0.40 (n° 112: CEPS n°82, CEPM n°30). In the latter condition stress echocardiography has been scheduled to evaluate the functional hemodynamic performance.

## Conclusions

A complete early Doppler-echocardiography evaluation of aortic prosthetic valves, including flow-dependent and independent Doppler echocardiography parameters, provides a baseline description of the prosthesis. Each patient who underwent aortic valve replacement should be supplied with Doppler echocardiography ID of the implanted prosthesis to allow an adequate interpretation of Doppler echocardiography examinations at follow-up.

## List of Abbreviations

AI: Aortic insufficiency; AS: aortic stenosis; AVR: Aortic valve replacement; BSA: Body Surface Area; CABG: coronary-aortic bypass grafting; CEPM: Carpentier-Edwards Perimount Magna; CEPS: Carpentier-Edwards Perimount Standard; CW: Continous-Wave; DVI: Doppler velocity index; EF: ejection fraction; EOA: effective orifice area; EOAi: indexed effective orifice area; LVOT: left ventricle outflow tract; PW: Pulse-Wave; PPM: patient-prosthesis-mismatch; VTI: velocity time integral.

## Competing interests

The authors declare that they have no competing interests.

## Authors' contributions

Concept/design: GM. Data analysis/interpretation: HP, MS, GP. Drafting article: GM. Critical revision of article: GM, FM., CG. Data collection: AP, ZG, FMO, DDS, MS, HP. All authors read and approved the final manuscript.

## Supplementary Material

Additional File 1 **Movie Clip 1: **CEPS aortic prostethic valve n°23 well functioning; Click here for file

Additional File 2 **Movie Clip 2: **CEPM aortic prostethic valve n°23 well functioning; Click here for file
